# Botulinum toxin treatment for essential palatal tremors presenting with nasal clicks instead of pulsatile tinnitus: a case report

**DOI:** 10.1186/s13005-016-0129-6

**Published:** 2016-11-22

**Authors:** Yufeng Ye, Shiyu Liao, Baozhen Luo, Liyan Ni

**Affiliations:** ENT Department, The Second Affiliated Hospital & Yuying Children’s Hospital of Wenzhou Medical University, 109 Xueyuan Western Road, Wenzhou, Zhejiang Province People’s Republic of China

**Keywords:** Case report, Palatal tremors, Botulinum toxin, Inferior olive, Pulsatile tinnitus, Electromyography

## Abstract

**Background:**

In this study, we report a rare case of an adult patient with essential palatal tremors (EPT) presenting as nasal clicks, instead of otic clicks or objective pulsatile tinnitus in common EPT.

**Case presentation:**

Nasal endoscopic examination and EMG recordings of the soft palate muscles were performed to confirm the source of the clicks. Initial treatment with lidocaine provided symptomatic relief for four hours. The patient was then treated with four simultaneous injections of 12.5 U of botulinum toxin in four different sites of the soft palate. Palatal tremors and clicks completely disappeared within three months of treatment.

**Conclusions:**

To our knowledge, this is the first case of EPT that presented with nasal clicks. We recommend that otolaryngologists should expect this rare occurrence in the clinical setting, and handle patients presenting with such symptoms with care and compassion in order not to worsen their psychological status.

**Electronic supplementary material:**

The online version of this article (doi:10.1186/s13005-016-0129-6) contains supplementary material, which is available to authorized users.

## Background

Palatal tremor i.s a rare movement disorder characterized by continuous rhythmic jerks of the soft palate that are often perceived as tinnitus [1]. Palatal tremors are classified as symptomatic palatal tremors (SPTs) and essential palatal tremors (EPTs). SPTs mainly present with neurological deficits such as dysarthria, nystagmus and ataxia. Abnormalities in the function of the nucleus dentatus, nucleus ruber and/or inferior olivary complex are known to contribute to the pathophysiology of SPT [2, 3]. SPTs rarely present with ear clicks and palatal movements, while EPTs almost exclusively present with ear clicks and palatal movements. Excessive contraction of the tensor veli palatini (TVP) muscle, which is innervated by the trigeminal nerve, is thought to be the cause of EPTs. According to the diagnostic criteria proposed by Deuschl et a1. [3, 4], no intracranial pathology is associated with EPTs. Patients have normal cerebellar function and pendular nystagmus. Furthermore, throat muscle involvement is not expected. In the literature, several cases of EPT associated with psychogenic factors, as well as those of unknown etiology, have been reported [5, 6].

Regardless of etiology, EPTs usually present with objective tinnitus of otic origin or ear clicks. However, EPTs with nasal clicks have hitherto not been reported. Here, we report the case of an adult patient with EPT presenting as nasal clicks who was effectively treated with botulinum toxin injection.

## Case presentation

### Patient history

A previously healthy 51-year-old man presented at our hospital with a chief complaint of clicking sounds heard in the nose. He mentioned that the noise was audible to others and disappeared during sleep. He had excessive phlegm for two years and pharyngalgia for one week. His birth, growth and developmental history were all normal. He had no history of head trauma, chronic ear disease, or neurological disorder. None of his family members had a history of nasal clicks or tinnitus. The patient had been very uncomfortable due to the persistent sound.

### Physical examination and preliminary diagnosis

An otolaryngologist performed physical examination of the nose, mouth and pharynx; and found that the audible “clicking” noise originated from the nose (Additional file 1: Video S1). No anomaly other than a fast rhythmic tremor of the soft palate was observed upon physical examination. Other muscles in the pharynx, mouth and eyes did not seem to be involved based on these examinations. Results of audiometry, tympanometry and neurologic examinations including cranial magnetic resonance imaging (MRI) and neurological physical examinations were all normal. Rhythmic palatal movements produced a high-pitched “clicking” sound with a frequency of approximately 100 clicks per minute (Additional file 1: Video S1, Additional file 2: Video S2 and Additional file 3: Video S3). The examiner could clearly hear the sound at a distance of 10 cm from the nasal tip. When the patient was asked to tilt his neck slightly backward, the noise was reduced (Additional file 4: Video S4).



**Additional file 1: Video S1.** “Clicking” noise and video recorded by cellphone. (MP4 1840 kb)




**Additional file 2: Video S2.** Oropharyngeal examination video recorded by endoscopy. (MP4 1700 kb)




**Additional file 3: Video S3.** Nasopharyngeal examination video recorded by endoscopy. (MP4 7560 kb)




**Additional file 4: Video S4.** Tremor ceased when tilted backward. (MP4 6310 kb)


**Fig. 1:**
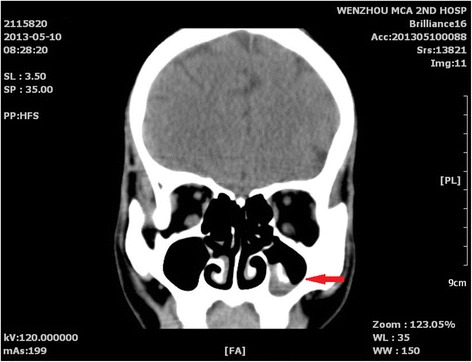
Coronal CT image of sinuses, indicating fungal rhinosinusitis of the left maxillary sinus

**Fig. 2:**
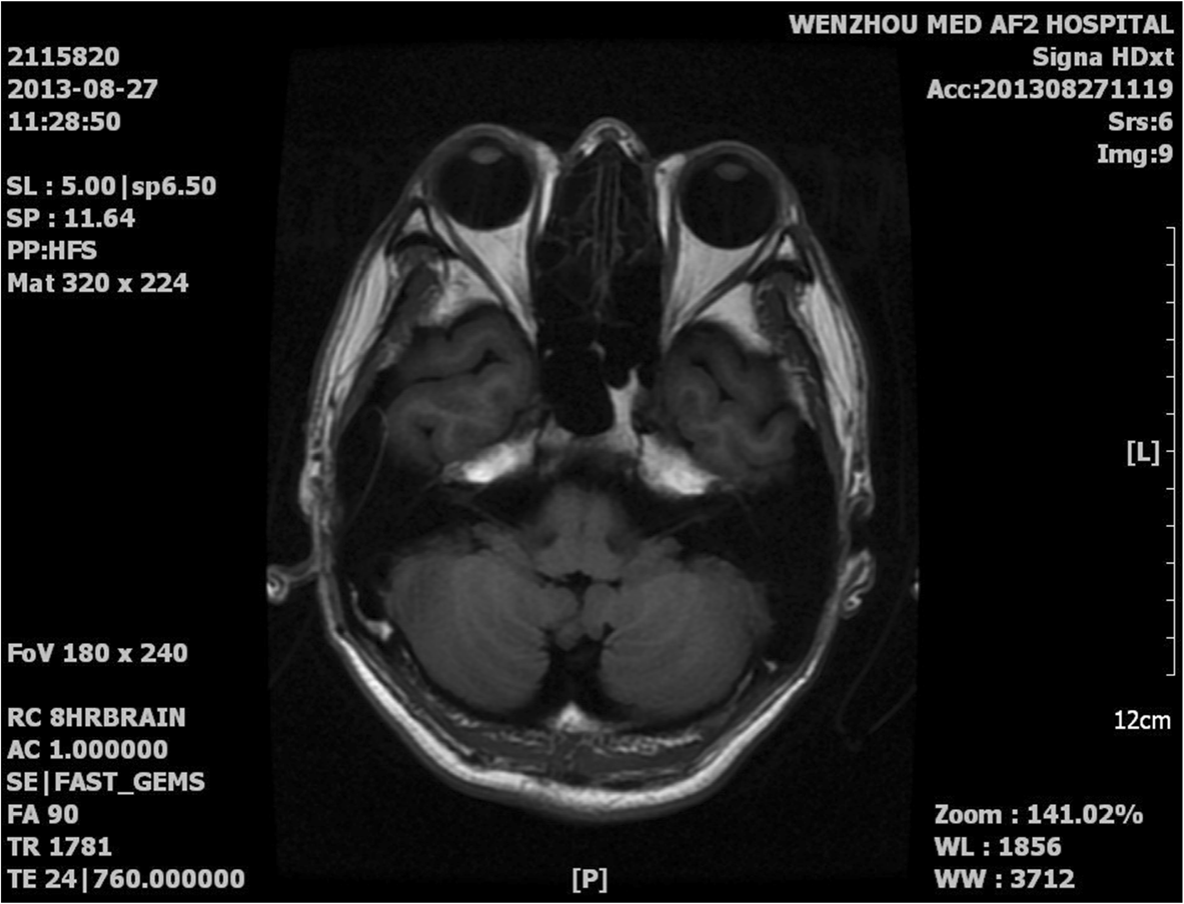
Cranial MRI image of the horizontal position. No occupying lesions or abnormal signal of the inferior olive was observed

Laboratory results including routine blood work, antinuclear antibody analysis, thyroid tests, and hepatic and renal functions tests were all normal (Table 1). Computed tomography (CT) scans of the brain and cervical region did not show any abnormalities. A nasal sinus CT scan revealed fungal maxillary sinusitis on the left side (Fig. 1), which was treated by functional endoscopic sinus surgery. No signs of olivary nucleus hypertrophy or occupying lesions of the skull base were visible on MRI scans (Fig. 2).

**Table 1:** Laboratory results including routine blood work, antinuclear antibody analysis, thyroid tests, and hepatic and renal functions

Test name	Abbreviations	Item name	Results	Ref	Unit	OD value
Hepatitis B virus	HBSAG	HbsAg	0.688 (Negative)	<1.000	COI	0.688
	AHBS	Anti-HBs	2.00 (Negative)		IU/L	2.000
	HBEAG	HbeAg	0.101 (Negative)	<1.000	COI	0.101
	AHBE	Anti-Hbe	1.56 (Negative)	>1.000	COI	1.560
	AHBC	Anti-HBc	1.89 (Negative)	>1.000	COI	1.890
HIV,HCV,TPHA	HIV	HIV antibodies (screen)	Negative	Negative		0
	HCV	Anti-HCV	Negative	Negative		0
	TPHA	Treponema pallidum specific antibody	Negative	Negative		0
Electrolytes, liver and kidney function	ALT	Glutamic-pyruvic transaminase	19	0 ~ 50	U/L	19.000
	AST	Glutamic oxalacetic transaminase	18	15 ~ 45	U/L	18.000
	AS/AL	AST/ALT	0.95			0.947
	ALP	Alkaline phosphatase	95	45 ~ 125	U/L	95.000
	GGT	Glutamyl transpeptidase	35	10 ~ 60	U/L	35.000
	TP	Total protein	78.9	61.0 ~ 79.0	g/L	78.900
	ALB	Albumin	44.9	34.0 ~ 48.0	g/L	44.900
	GLO	Globulin	34.0	24.8 ~ 38.8	g/L	34.000
	A/G	ALB/GLB	1.3	1.2 ~ 2.0		1.321
	TBIL	Total bilirubin	10.4	6.8 ~ 34.2	μmol/L	10.400
	DBIL	Direct bilirubin	1.5	1.7 ~ 8.6	μmol/L	1.500
	IBIL	Indirect bilirubin	8.9	4.8 ~ 25.0	μmol/L	8.900
	GLU-S	Fasting blood-glucose	5.53	3.90 ~ 6.10	mmol/L	5.530
	BUN	Serum urea	3.82	2.90 ~ 8.20	mmol/L	3.820
	CREA	Creatinine	91.2	50.0 ~ 133.0	μmol/L	91.200
	BU/CR	Serum urea/Creatinine	0.042	0.03 ~ 0.15		0.042
	UA	Uric acid	260	149 ~ 416	μmol/L	260.000
	NA	Sodium	137.5	137.0 ~ 147.0	mmol/L	137.500
	K	Kalium	4.33	3.50 ~ 5.30	mmol/L	4.330
	CL	Chlorine	105.3	99.0 ~ 110.0	mmol/L	105.300
Routine stool test	YS	Color	Yellow			0
	JD	Toughness	Soft			0
	NY	Mucus	Negative			0
	BXQ	White blood cells (stool)	Negative		/High power field	0
	HXB	Red blood cells (stool)	Negative		/High power field	0
	NC	Pyocyte	Not found		/Low power field	0
	AMB	Amebic protozoa	Not found	Not found		0
	JMJ	Saccharomycopsis	Not found	Not found		0
	HCL	Ova of roundworm	Not found	Not found		0
	NX	Purulent blood	Negative	Negative		0
	CL	Ovum	Not found	Not found		0
	JSC	Parasite	Not found			0
Urine routines	COLOR	Color	Faint yellow			2.000
	TURB	Turbidity	Clear			0
	WBC	Leukocyte count	0.54		/High power field	0.540
	WBC1	Leukocyte count	3.00		/μl	3.002
	RBC	Erythrocyte count	1.80		/High power field	1.800
	RBC1	Erythrocyte count	10.01		/μl	10.008
	TMGX	Hyaline cast	2.90		/Low power field	2.900
	WFLGX	Granular casts	Negative	Negative	/Low power field	−2.900
	CG	Cellular cast	Negative	Negative	/Low power field	−2.900
	LZSP	Squamous epithelial cell	5.80		/Low power field	2.000
	BLD	Urine occult blood	Weakly positive (±)	Negative		0
	BIL	Urine bilirubin	Negative	Negative		0
	URO	Urobilinogen	Negative	Negative		0
	KET	Urine acetone bodies	Negative	Negative		0
	PRO	Qualitative test of urinary protein	Weakly positive (±)	Negative		0
	SG	Urine specific gravity	1.019	1.003 ~ 1.03		1.019
	NIT	Nitrite	Negative	Negative		0
	GLU	Urine sugar	Negative	Negative		0
	PH	pH	7.0	4.6 ~ 8		7.000
	LEU	Neutrophil esterase	Negative	Negative		0
Blood coagulation	PT	Prothrombin time	12.00	12.00 ~ 15.00	Second	12.000
	PTS-CP	Normal controls (PT)	12.50		Second	12.500
	INR	International normalized ratio	0.95	0.85 ~ 1.15		0.950
	APTT	Activated partial thromboplastin time	32.10	30.00 ~ 45.00	Second	32.100
	APTTS-	Normal controls (APTT)	35.00		Second	35.000
	TT	Thrombin time	16.30	14.00 ~ 18.00	Second	16.300
	FIB	Fibrinogen	2.95	2.00 ~ 4.00	g/L	2.950
	BBBC	Sample preservation	The sample retained for three days			1.000
Blood routine	WBC	Leukocyte count	7.00	4 ~ 10	×10^9/L	7.000
	NEUT%	Ratio of neutrophil	0.647			0.647
	LYMPH%	Ratio of lymphocyte	0.286			0.286
	MONO%	Ratio of monocytes	0.044			0.044
	EO%	Ratio of eosinophils	0.013			0.013
	BASO%	Ratio of basophils	0.006			0.006
	IG%	Ratio of immature granulocytes	0.004			0.004
	YC1	Annormal lymphocytes	Not found			0
	YZXB5	Juvenile cells	Not found			0
	NEUT#	Neutrophil count	4.529		×10^9/L	4.529
	LYMPH#	Lymphocyte count	2.002		×10^9/L	2.002
	MONO#	monocyte count	0.308		×10^9/L	0.308
	EO#	eosinophil count	0.091		×10^9/L	0.091
	BASO#	Basophil count	0.042		×10^9/L	0.042
	IG#	immature granulocyte count	0.028		×10^9/L	0.028
	HGB	Hemoglobin	148	120 ~ 160	g/L	148.000
	RBC	Erythrocyte count	4.88	4 ~ 5.5	×10^12/L	4.880
	HCT	Hematokrit	0.429	0.4 ~ 0.54		0.429
	MCV	Mean corpuscular volume	87.90	80 ~ 100	fl	87.900
	MCH	Mean corpuscular hemoglobin	30.30	27 ~ 34	pg	30.300
	MCHC	Mean corpuscular-hemoglobin concentration	345	320 ~ 360	g/L	345.000
	RDW-CV	Red cell distribution width-CV	13.0	11.6 ~ 14.6	%	13.000
	RDW-SD	Red cell distribution width-SD	41.5		fl	41.500
	PLT	platelets counts	334	100 ~ 300	×10^9/L	334.000
	PCT	Thrombocytocrit	0.34	0.11 ~ 0.28		0.340
	MPV	Mean platelet volume	10.30	6.5 ~ 11	fl	10.300
	P-LCR	Platelet-large cell ratio	27.40		%	27.400
	PDW	Platelet distribution width	12.30	9 ~ 17	%	12.300
	ZDKL	Toxic granulation	Negative			0
	YHH	Nucleated red blood cell count	0.0		/100WBC	0
	YCC	Abnormal cell	Not found			0
	BBBC	Sample preservation	The sample retained for three days			1.000
Blood type	ABO	ABO blood group system	AB			0
	RH	RH(D) blood group	Positive			0
Thyroid function tests	TOTT3	Total T3	0.78	0.87 ~ 1.78	ng/ml	0.780
	TOTT4	Total T4	7.01	6.10 - 12.20	μg/dl	7.010
	FT3	Free T3	2.52	2.50 ~ 3.90	pg/ml	2.520
	FRT4	Free T4	0.88	0.61 ~ 1.12	ng/dl	0.880
	TSH	Thyroid stimulating hormone	1.06	0.34 ~ 5.60	μIU/ml	1.060
Bronchofiberscope		Mould	Not found			0
		Smear results	Found no acid fast bacilli			0

### Nasal endoscopic examination and sinus surgery

We then performed a nasal endoscopic examination. Involuntary rhythmic movements of the nasopharyngeal lateral wall or muscles surrounding the eustachian tube were not observed (Additional file 2: Video S2 and Additional file 3: Video S3). However, the torus tubarius was found to be involved. We identified the source of the clicks on the basis of the adjacency of the sound to the nose and throat. Thus, the nasal cavity was confirmed as the source of the sound.

Endoscopic sinus surgery was performed to open the left maxillary sinus. The patient was placed in the supine position. After endotracheal intubation and routine disinfection, sterile drapes were placed and the operation commenced. An adrenaline cotton sheet was placed in the left nasal mucosa to absorb nasal secretions. The middle turbinate root, agger and uncinate process of the left side were locally anesthetized by injecting lidocaine-containing adrenaline. Under 0° endoscopy, the left uncinate process was lifted by a nasal probe, and the tail section of the uncinate process was separated. The upper and lower ends of the uncinate process adjacent to the lateral nasal wall were excised with a curved scissor. The separated uncinate process was removed with an ethmoidal sinus forcep. Thereafter, the bone at the tail end of the uncinate process was removed with a detacher and the anterior fontanelle was removed with a back biting rongeur. Thus, the natural orifice of the maxillary sinus was expanded. The maxillary sinus observed with 70° endoscopy revealed several brown bean-curd-residue-like lesions. A subsequent pathological examination confirmed that they were mould clumps. We cleaned the lesions, flushed the maxillary sinus cavity with saline via a curved suction tube, and ensured that the sinus mucosa was smooth and without any residual lesions. The left nasal cavity was filled with one expansion sponge, and the operation was completed.

However, the “clicking” noise did not stop. In a follow-up exam three months after surgery, we confirmed that the ostium of the left maxillary sinus had significantly opened up, and the sinus was clean.

### Electromyography

**Fig. 3:**
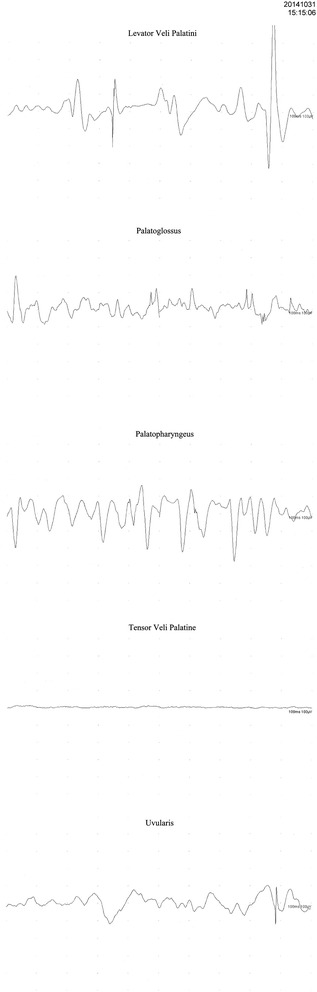
EMG recordings of palatal tremor activities are shown

To determine the etiology of the nasal click, we recorded the activities of the soft palatal muscles including the TVP, palatopharyngeus, palatoglossus, levator veli palatini (LVP), and uvularis muscles by direct electromyography (EMG, Fig. 3). All the above-mentioned muscles except the TVP revealed abnormal waveforms. This was a highly unusual finding. The TPV is usually the main muscle associated with EPT. However, our EMG results revealed that all other muscles except the TVP were causing these contractions.

### Treatment

**Fig. 4:**
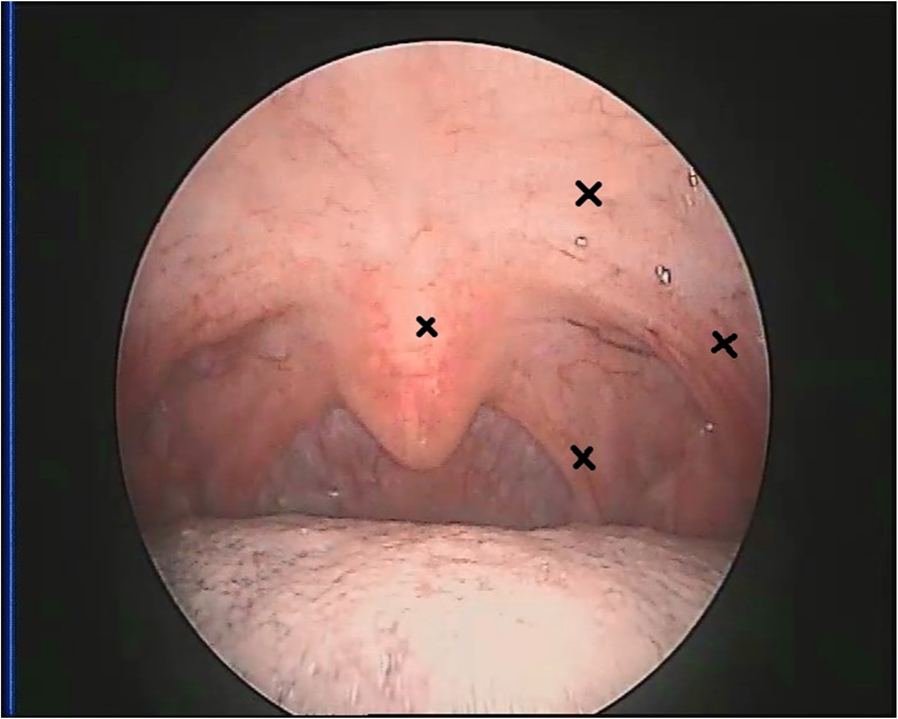
Sites of botulinum toxin injections



The nasal clicks significantly affected the patient’s social life. The patient did not receive any other treatment from other hospitals before he arrived at our hospital. To explore whether blocking nerve impulse conduction can weaken or eliminate the tremors, we first injected 0.5 mL of 2% lidocaine into the palatal muscles including the palatopharyngeus, palatoglossus, LVP and uvularis muscles. Palatal tremors disappeared, but returned within four hours given that the effective time of lidocaine anesthesia is approximately 1.5 h. When the patient visited our hospital a week later, we modified the treatment strategy. Botulinum toxin was injected into four different regions (dose, 12.5 U/site) of the soft palatine (Fig. 4). The patient tolerated the injections well on the first day. On the second day, the patient experienced dragging pain, and his tongue twisted involuntarily when he had to open his mouth wide. Two weeks later, the pain subsided, and his lisp disappeared. The patient did not complain of excessive phlegm thereafter. No other side effects such as choking after drinking or difficulty in swallowing were reported. Three months after receiving botulinum toxin injections, the palatal tremors disappeared; and the patient felt well. However, eight months after the botulinum toxin injections, the palatal tremors recurred; but the frequency of the tremors and number of attacks were reduced. Rapid contractions of the velar muscles sometimes occurred when he was nervous or tired, or had consumed alcohol; but he could control them voluntarily (Additional file 5: Video S5). An EMG test at this point showed complete relief of the LVP and uvularis muscles besides palatoglossus and palatopharyngeus.

**Additional file 5: Video S5.** Endoscopic record 8 months after injection. (MP4 22200 kb)


## Discussion

Auditory clicks arising from the rhythmic contraction of any of the muscles in the ear and throat [1, 2] are regarded as a primary symptom of EPT. The case of our patient was unique, because the clicks were nasal in origin.

The tremors would stop when the patient was asleep. The palatal tremor cycle could not be restored by inhibition of the trigeminal nerve using lidocaine. Neurological examination results and brain MRI scans were normal. MRI scans have shown increased signal intensity on T2 weighted images in SPT patients with injury to the dentate-olivary complex, indicating hypertrophy of the olivary nucleus [7, 8]. MRI scans of our patient revealed no evidence of structural abnormalities. Thus, SPT was excluded as a cause. To our knowledge, this is the first case of EPT associated with nasal clicks instead of otic clicks.

Otic clicks are caused by abnormal contractions of both the TVP and LVP muscles. However, objective otic clicks due to LVP contractions have not been reported since 1996 [9]. In our patient, otic clicks were completely absent (Additional file 1: Video S1). Therefore, we suspected that some muscles of the soft palate other than the TVP such as the palatopharyngeus, palatoglossus, uvularis, and/or LVP muscles contributed to the nasal clicks. EMG results confirmed that all the above muscles were involved, except the TVP.

EPT clicks are affected by mouth opening, speaking [9], head position [10] and relaxation [11]. In our patient, tilting the head backwards and speaking could completely suppress the tremors.

No specific treatment has been reported for EPT. In 1997, Cakmur et al. [12] reported that a 16-year old girl, who had been diagnosed with EPT at the age of six, was successfully treated with flunarizine, which is a selective calcium entry blocker with antihistaminic, antiserotoninergic and antidopaminergic activity [13]. However, the tremor was found to recur after flunarizine was discontinued.

Campistol-Plana et al. [14] suggested that TPV and tubal pharynx muscles are controlled by the glossopharyngeal nerve and pharyngeal plexus. Their treatment of four pediatric patients with 2% lidocaine resulted in the gradual disappearance of palatal tremors.

Nasr and Brown reported that a 37-year-old man with a history of alcohol abuse, palatal tremors and ear clicks, and who had been hospitalized for excessive alcohol intake, has shown gradual improvement of ear clicks and frequency of tremors after being treated with lamotrigine, which is a sodium channel blocking antiepileptic drug [15]. However, the long-term success of this drug could not be determined, because the patient discontinued its use after being discharged from the hospital.

Botulinum toxin is a neurotoxin that blocks neuromuscular transmission by inhibiting the acetylcholine receptor. Since the establishment of botulinum toxin as a safe and tolerable treatment for various therapeutic indications [16] including migraine [17], cerebral palsy [18], and cervical and maxillofacial conditions [19], it has been sporadically tested for treating EPTs.

In some cases, botulinum toxin was directly injected into the soft palate to ameliorate symptoms [20, 21]. Its efficacy in EPT stems from the fact that it binds to cholinergic nerve endings and causes muscular paralysis, thereby reducing muscular contractions. Its long-term use has not been found to cause permanent muscular degeneration [22, 23].

Recently, Tobias et al. [24] reported that long-term botulinum toxin injections could relieve objective tinnitus in a 78-year-old woman with minimal side effects. However, the toxin yielded temporary effects and had to be administered every 5–6 months to prevent recurrence of symptoms that could affect the daily activities of the patient.

Anis and Pollak reported that a 36-year-old woman with EPT, who had failed to respond to conservative treatment with anxiolytics, was successfully treated with 2–3 injections of botulinum toxin [25]. Symptomatic relief was obtained within two days of injection.

Even though the source of the clicks in our patient was different from that reported in previous cases, we applied symptom-guided injections of botulinum toxin in our patient. Unlike the temporary relief provided by lidocaine injections, four simultaneous injections of botulinum toxin were found to diminish the tremors in our patient. Although our patient tolerated the treatment well, alleviation of symptoms required at least two weeks. Variability in time and dosage required for symptomatic relief in our patient and in patients reported in previous studies suggests that individualized titration of dose and frequency by close monitoring of symptoms may be critical in achieving long-term benefits, as suggested by Anis and Pollak [25].

Although the etiology of EPT remains unclear, some patients were found to have minor ailments [26] or symptoms such as otitis media, fever, or tonsillitis before the occurrence of EPTs. Our patient had excessive phlegm for two years and pharyngalgia for one week. We believe that these symptoms could be related to the etiology of EPT, because they were spontaneously relieved within 15 days after botulinum toxin treatment. Additional studies will be required to determine the pathogenesis of EPT with nasal clicks, as well as the occurrence of phlegm and pharyngalgia.

## Conclusions

Tinnitus is the most common symptom of EPT. In this study, we report the first case of a patient with EPT who presented with nasal clicks, instead of objective pulsatile tinnitus. Similar to previous reports, botulinum toxin injections were found to alleviate symptoms and improve the quality of life of the patient for up to three months. Although the detailed pathophysiology and etiology of EPT that presented with nasal clicks remains unclear, we recommend that otolaryngologists should expect this rare occurrence in the clinical setting. Patients with this rare disorder usually have a poor social life. Therefore, clinicians should exercise care, patience and attention when handling such cases in order not to worsen the psychological status of the patient.

## Abbreviations

CT: Computed tomography
EMG: Electromyography
EPT: Essential palatal tremor
LVP: Levator veli palatini
MRI: Magnetic resonance imaging
SPT: Symptomatic palatal tremor
TVP: Tensor veli palatini
